# Salt stress induced differential metabolic responses in the sprouting tubers of Jerusalem artichoke (*Helianthus tuberosus* L.)

**DOI:** 10.1371/journal.pone.0235415

**Published:** 2020-06-29

**Authors:** Hui-Xi Zou, Dongsheng Zhao, Haihong Wen, Nan Li, Weiguo Qian, Xiufeng Yan

**Affiliations:** Zhejiang Provincial Key Laboratory for Subtropical Water Environment and Marine Biological Resources Protection, College of Life and Environmental Science, Wenzhou University, Wenzhou, Zhejiang, People’s Republic of China; Northeast Forestry University, CHINA

## Abstract

To better understand the mechanism of inherent salt resistance in Jerusalem artichoke (*Helianthus tuberosus* L.), physiological and metabolic responses of tubers at the initiation stage of sprouting under different salt stress levels were evaluated in the present study. As a result, 28 metabolites were identified using proton nuclear magnetic resonance (^1^H-NMR) spectroscopy. Jerusalem artichoke tubers showed minor changes in metabolic response under moderate salt stress when they had not yet sprouted, where metabolism was downregulated at the start of sprouting and then upregulated significantly after plants became autotrophic. However, mild and severe salt stress levels caused different metabolic response patterns. In addition, the accumulation of fructose and sucrose was enhanced by moderate salt stress, while glucose was highly consumed. Aspartate and asparagine showed accelerated accumulation in sprouting Jerusalem artichoke tubers that became autotrophic, suggesting the enhancement of photosynthesis by moderate salt stress.

## Introduction

Developing a new plant individual from a propagule (seed or tuber) is the most important developmental stage in the whole life cycle of plants, which are confronted with the perception of and adaptation to the ecological environment for supporting seedling survival, especially in unfavorable environmental conditions. Understanding the dynamic process of physiological and metabolic changes at the initiation stage of plant growth will be critical for determining the mechanisms of plant responses to environmental stress. The accumulation of salts in soil affects more than 400 million hectares of land worldwide (The Food and Agriculture Organization, 2002). Approximately 12 million hectares of cultivated field are facing the challenge of soil salinization [1,2]. Determining sources of salinity-resistant plants is of great importance for responding to the growing ecological problem of increasing salinity in soil.

Jerusalem artichoke (*Helianthus tuberosus* L.) is an annual sunflower plant that is cultivated as a vegetable, fodder crop and bioenergy material due to its high tuber biomass and enrichment of inulin [3–5]. The high ecological value of Jerusalem artichoke is that it can survive and even grow well in saline-alkali soils [6]. Several strategies, e.g., osmotic adjustment, ion selective absorption and antioxidant system reinforcement, have been applied to adapt Jerusalem artichoke to saline environments [7–9], and they are common physiological mechanisms by which halophytes respond to salt stress. Jerusalem artichokes are propagated from tubers that represent the primary choice for commercial production. However, there have only been occasional reports of successful production of the crop from seed [10]. Moreover, seedling establishment of Jerusalem artichoke would be severely repressed in high-salinity fields if seeds were used rather than tubers. The possible explanation might be the various storage carbohydrates in the tubers of Jerusalem artichoke that enable seedlings to thrive under salt stress [11,12]. Considering the variety of tuber resources, we presumed that there might be a specific mechanism that allows for Jerusalem artichoke propagation from tubers with inherent salt tolerance.

Our previous study generated a composite, well-annotated transcriptome and proteome of Jerusalem artichoke roots in response to salt stress, which suggested that the induced activity of ribosome and sugar signaling may provide Jerusalem artichoke with salt tolerance [13]. Jerusalem artichokes are tubers of plants that form at the base of roots and store energy in the form of starch to support new stem and root growth. Moreover, different mechanisms may be employed in different developmental stages of plants, especially those under environmental stress, resulting in different metabolic responses. Thus, the metabolome response in sprouting tubers of Jerusalem artichoke subjected to different salt stress levels was evaluated using proton nuclear magnetic resonance (^1^H-NMR) spectroscopy in the present study. The metabolite profiles of tubers at the sprout emergence stage were examined to describe metabolic reprogramming under salt stress and explain the modulated functions of specific metabolic pathways in Jerusalem artichoke in response to high salinity. Determining the dynamic response process of Jerusalem artichoke during tuber sprouting could help us understand the physiological mechanism of inherent salt resistance in the tubers.

## Materials and methods

### Plant material, growth conditions, stress treatment

Jerusalem artichoke tubers were collected from naturally saline agricultural land in Zhaodong, Heilongjiang Province, China. Plant tubers were sown in plastic pots filled with aseptic vermiculite and fertile black soil (2:1, v/v) and grown in a greenhouse at 25/20°C (day/night) with an 8 h light/16 h dark photoperiod, photosynthetically active radiation at 150 mol m^−2^ s^−1^ and relative humidity at 50–70%. All the planted tubers were separated into two sets. One set was irrigated daily by half-strength Hoagland’s solution (pH 6.21 ± 0.10) with or without 50, 150 or 250 mM NaCl for 7 days, and another set was treated with 150 mM NaCl for 1 day, 3 days and 7 days. The tubers from different groups were washed with distilled water, followed by surface drying with filter paper. After quantifying the growth phenotype, sprouts and roots were removed from tubers. Tuber tissues were cut into small pieces, frozen in liquid nitrogen and stored at -80°C until further analysis.

### Measurement of biomass

The lengths of the sprouts and roots were recorded, as well as the relative water content (RWC). For each measurement, four individual plants were used. Samples were harvested after exposure and washed with distilled water, followed by surface drying with filter paper. After the determination of fresh weight (FW), the samples were dried in an oven at 80°C to a constant weight, and then the dry weight (DW) was determined. RWC was calculated as (FW-DW)/FW×100%.

### Metabolomic analysis

#### Metabolite extraction

Polar metabolites were extracted from tubers using the solvent system of methanol/water (1/1) and a modified extraction protocol as described previously [14,15]. Ten replicates were used for each treatment. Fresh leaf tissue (approximately 100 mg) was ground in liquid nitrogen with a mortar and pestle. The tissue powder was transferred to a tube containing approximately 50 ceramic beads with a 1 mm diameter and homogenized in 3.33 mL g^−1^ methanol/water (1/1). After vortexing for 15 s three times, the homogenate was centrifuged at 3,000×g for 10 min at 4°C, and the supernatant was removed and then lyophilized. the residue was subsequently resuspended in 600 μL phosphate buffer (0.1 M Na_2_HPO_4_ and NaH_2_PO_4_, including 0.5 mM TSP; pH 7.0) in D_2_O. Before being centrifuged at 3,000×g for 5 min at 4°C, the mixture was vortexed for 15 s, and then the supernatant (550 μL) was pipetted into a 5 mm NMR tube for subsequent NMR analysis.

#### NMR analysis

Metabolite extract analysis of Jerusalem artichoke tubers was performed with a Bruker AV 500 NMR spectrometer at 500.18 MHz (at 25°C, 298 K) [16]. One-dimensional (1D) ^1^H-NMR spectra were obtained with the following parameters: 11.9 μs pulse, 6,009.6 Hz spectral width, 0.1 s mixing time, and 3.0 s relaxation delay with standard 1D NOESY pulse sequence, with 128 transients collected into 16,384 data points. Datasets were zero-filled to 32,768 points, and exponential line-broadenings of 0.3 Hz were applied prior to Fourier transformation.

#### Spectral processing and multivariate data analysis

All ^1^H-NMR spectra were phased, baseline-corrected, and calibrated (TSP at 0.0 ppm) using TopSpin (version 2.1, Bruker). Metabolites were assigned and quantified following the tabulated chemical shifts [17,18] and processed using custom-written ProMetab software in MATLAB (version 7.0; The MathWorks, Natick, MA, USA), as reported previously [19,20]. Bins with widths of 0.005 ppm between 0.2 and 10.0 ppm were segmented from each spectrum. The bins of the residual water peaks between 4.70 and 5.20 ppm were excluded from the NMR spectra. Data were mean-centered before multivariate analysis. PCA was used in this work to display the similarities and variations between multiple NMR spectra from different samples. The PCA algorithm calculates the highest amount of correlated variation along principal component 1 (PC1), with subsequent PCs containing correspondingly smaller amounts of variance. For each model built, the loading vectors for the PCs could be used for the identification of the contributive metabolites (metabolic biomarkers) for the clusters.

Multivariate data analysis was performed using SIMCA-P^+^ software (Version 11.0, Umetric, Sweden). O-PLS-DA was subsequently carried out to determine the statistically significant metabolite responses to different cases of salt treatment. Score plots were generated to show the classifications, and corresponding loading plots were generated to describe the NMR spectral variables contributing to the classifications. The model coefficients were calculated from the coefficients incorporating the weight of the variables to enhance interpretability of the model. Then, metabolic differences responsible for the classifications between the control and salt-treated groups were detected in the coefficient-coded loading plots generated by using MATLAB with an program developed in-house. The variations were color-coded by absolute values of coefficients (r). Red corresponded to metabolites that were highly positively or negatively significant in discriminating between groups, and blue corresponded to no significance. The test of the significance of Pearson’s product-moment correlation coefficient was used to determine the correlation coefficient. The validation of the model was conducted using 6-fold cross validation, and the cross-validation parameter Q^2^ was calculated. A permutation test (n = 200) was also conducted to evaluate the validity of the PLS-DA models [21,22]. The R^2^ in the permutated plot described how well the data fit the derived model, whereas Q^2^ described the predictive ability of the derived model and provided a measure of the model quality. If the maximum value of Q^2^ max from the permutation test was smaller than or equal to the Q^2^ of the real model, the model was regarded as a predictable model. Similarly, the R^2^ value and difference between R^2^ and Q^2^ were used to evaluate the possibility of overfitted models.

Data were statistically analyzed using SPSS 13.0. Six replications were conducted for each treatment group, and the means and calculated standard deviation (SD) were reported. Tukey’s HSD test was performed to test the statistical significance (*p* < 0.05 and *p* < 0.01) of differences between the control and treated groups.

## Results

### General growth performance under different salt stress levels

**Fig 1:**
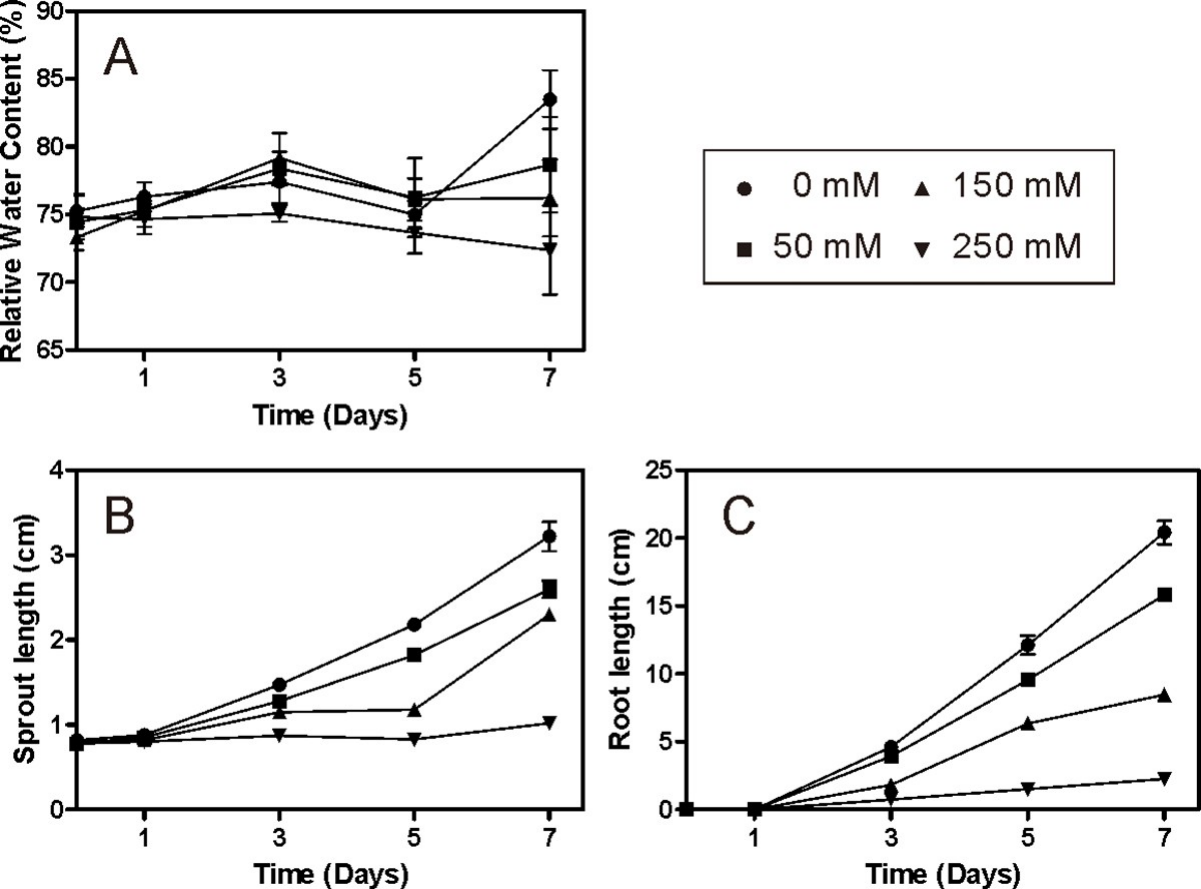
Effect of salt stress on Jerusalem artichoke tuber spouting. (A) Relative water content (RWC) of tuber (%). (B) Sprout length (cm). (C) Root length (cm) (Mean ± SE, n = 4).

The growth of both sprouts and roots under different salt stress levels is summarized in Fig 1, which shows inhibition effects of salinity on the growth rate of Jerusalem artichoke tubers. Root formation proceeded in parallel with sprout development in all treatments, both of which were NaCl concentration-dependent. Consistent with our previous study, a drastic effect was also observed in Jerusalem artichoke tubers under 250 mM NaCl stress (severe salt stress) compared to the control [22]. For the other two salt-treated groups as well as the control group significant growth in both sprouts and roots was observed after 3 days, indicating the start of sprouting. Furthermore, an obvious increase in relative water content was only observed in the control group after 7 days. Based on phenotypic observations and the results of our previous study, to obtain relatively healthy plants that display a salt-resistant phenotype, we chose 150 mM NaCl-treated (moderate salt stress) plants for metabolomics analysis. In addition, the metabolic responses of plants subjected to different salt stress levels (50 and 250 mM NaCl) were also investigated.

### Metabolic responses

**Fig 2:**
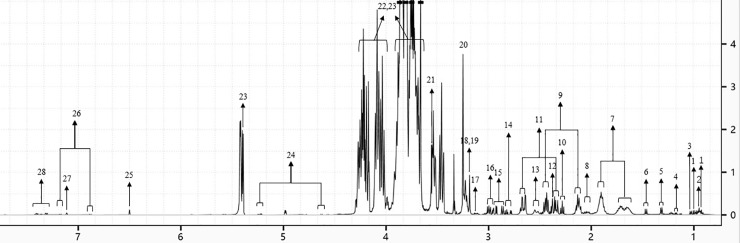
A representative ^1^H-NMR spectrum of the metabolites identified in Jerusalem artichoke tubers. (1) isoleucine, (2) leucine, (3) valine, (4) ethanol, (5) threonine, (6) alanine, (7) arginine, (8) glutamate, (9) glutamine, (10) 4-aminobutyrate, (11) malate, (12) succinate, (13) citrate, (14) aspartate, (15) asparagine, (16) 2-oxoglutarate, (17) malonate, (18) choline, (19) O-phosphocholine, (20) betaine, (21) glycine, (22) fructose, (23) sucrose, (24) glucose, (25) fumarate, (26) tyrosine, (27) histidine, (28) phenylalanine.

A total of 28 different metabolites were detected and quantitated by ^1^H-NMR, including 14 amino acids (isoleucine, leucine, valine, threonine, alanine, arginine, glutamate, glutamine, aspartate, asparagine, glycine, tyrosine, histidine, and phenylalanine), 7 organic acids (malic, succinic, citric, malonic, 2-oxoglutaric, and fumaric acids and γ-aminobutyric acid (GABA)), 3 sugars (fructose, sucrose, and glucose) and 4 other compounds (choline, phosphocholine, betaine, and ethanol), as shown in Fig 2. The ^1^H-NMR spectra was dominated by soluble sugars, which play an important role in energy metabolism and osmotic regulation under salt stress.

**Fig 3:**
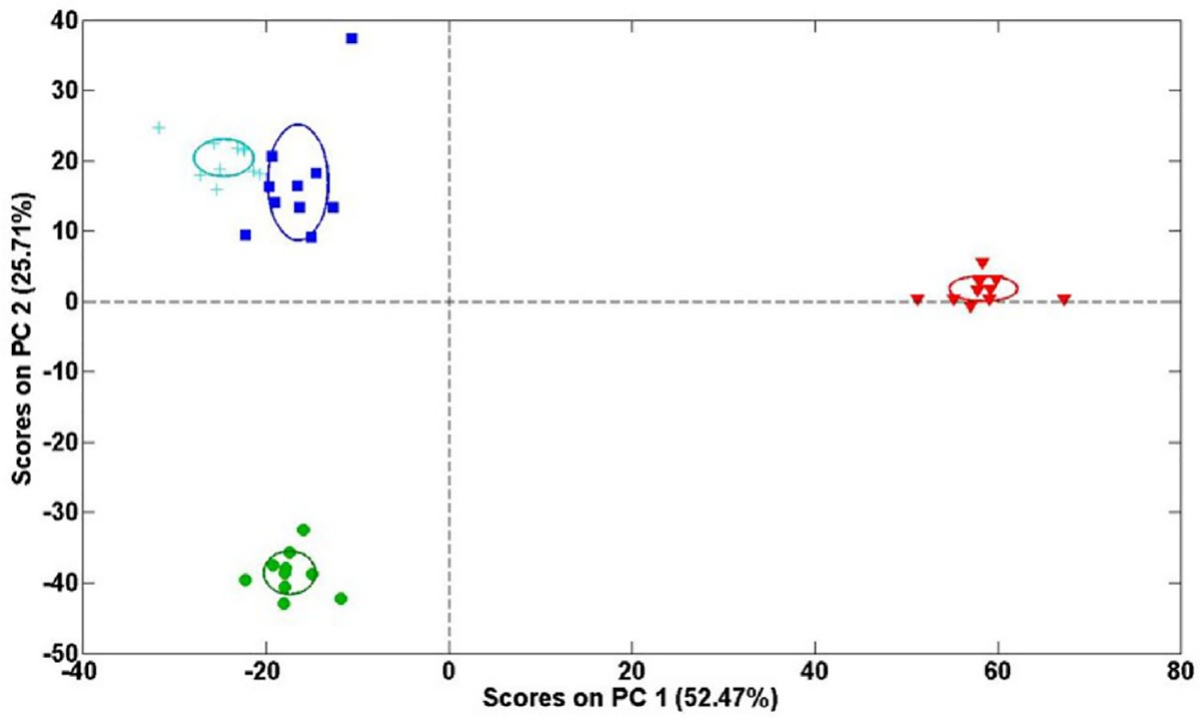
Score plots of Principal Component Analysis (PCA) of metabolite profiles from Jerusalem artichoke. Untreated (light blue cross) and treated with 150 mM NaCl for 1 day (blue square), 3 days (green circle) and 7days (red inverted triangle). Each sample of different groups presented by a single data point. PC1 and PC2 covering 78.18% of the variance. The variable salt treatment contributions for the clustering of metabolite samples.

**Fig 4:**
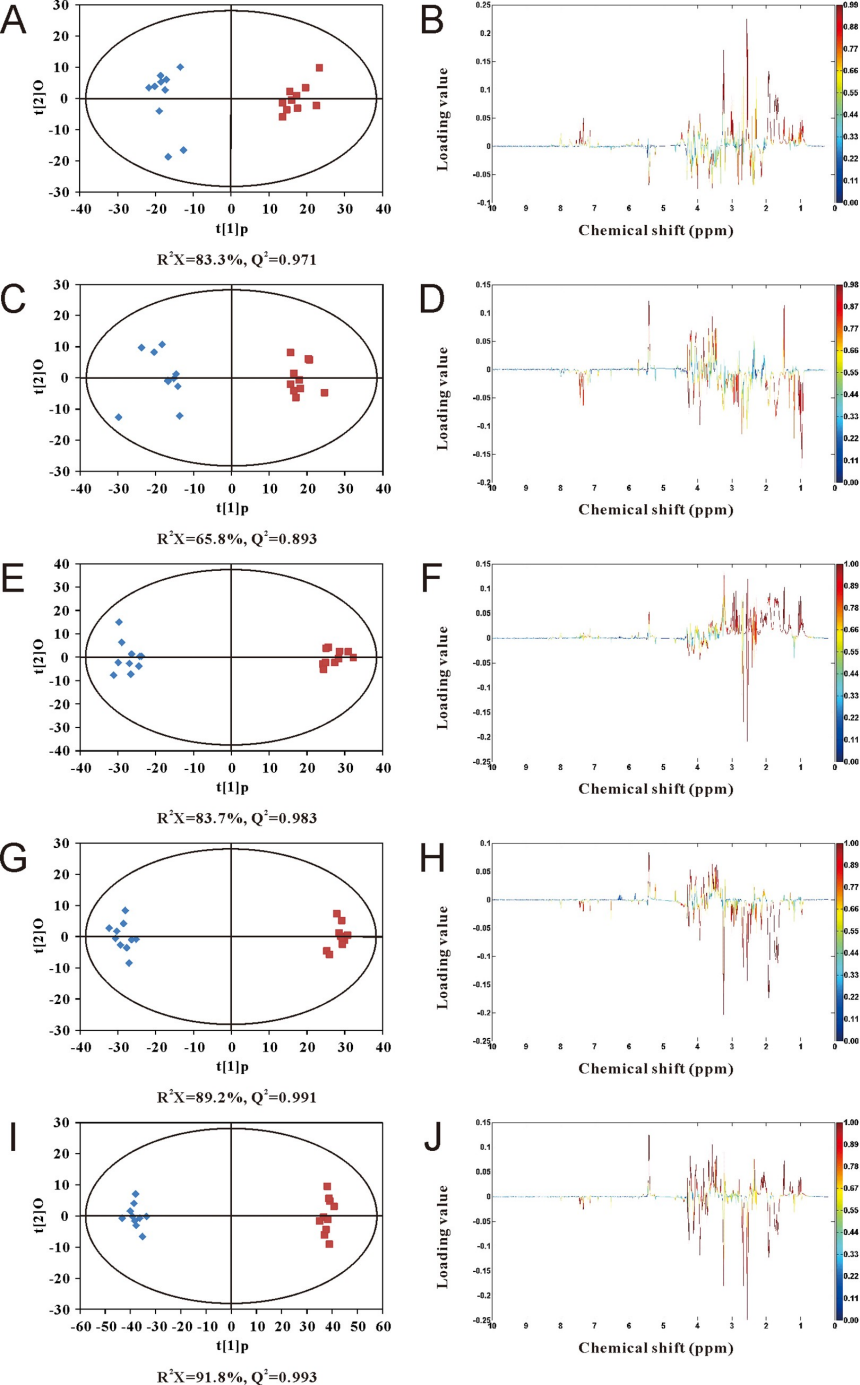
O-PLS-DA score plots derived from ^1^H-NMR spectra of extracts from Jerusalem artichoke tubers. (A, C, E, G and I) O-PLS-DA score plots. (B, D, F, H and J) Coefficient plots. (A) and (B) 150 mM NaCl, 1 day. (C) and (D) 150 mM NaCl, 3 days. (E) and (F) 150 mM NaCl, 7 days. (G) and (H) 50 mM NaCl, 7 days. (I) and (J) 250 mM NaCl, 7 days. The color map shows the significance of metabolite variations between the two classes (♦ control and ■ salt-treated). Peaks in the positive direction indicate metabolites that are more abundant in the salt-treated group. Consequently, metabolites that are more abundant in the control group are presented as peaks in the negative direction.

Principal component analysis (PCA) was conducted with the ^1^H-NMR spectral datasets to summarize the similarities and differences between samples of Jerusalem artichoke under moderate salt stress for 1, 3 and 7 days. The score plots are shown in Fig 3, where PC1 and PC2 explained 52.47% and 25.71% of the variation, respectively, separating the samples into three distinct groups. Furthermore, orthogonal projection to latent structure with discriminant analysis (O-PLS-DA) was performed on the ^1^H-NMR spectral datasets from the control and salt-treated groups. The score plots (Fig 4) derived from O-PLS-DA indicated clear distinction of the control and salt-treated groups, with reliable Q^2^ values (>0.8).

**Fig 5:**
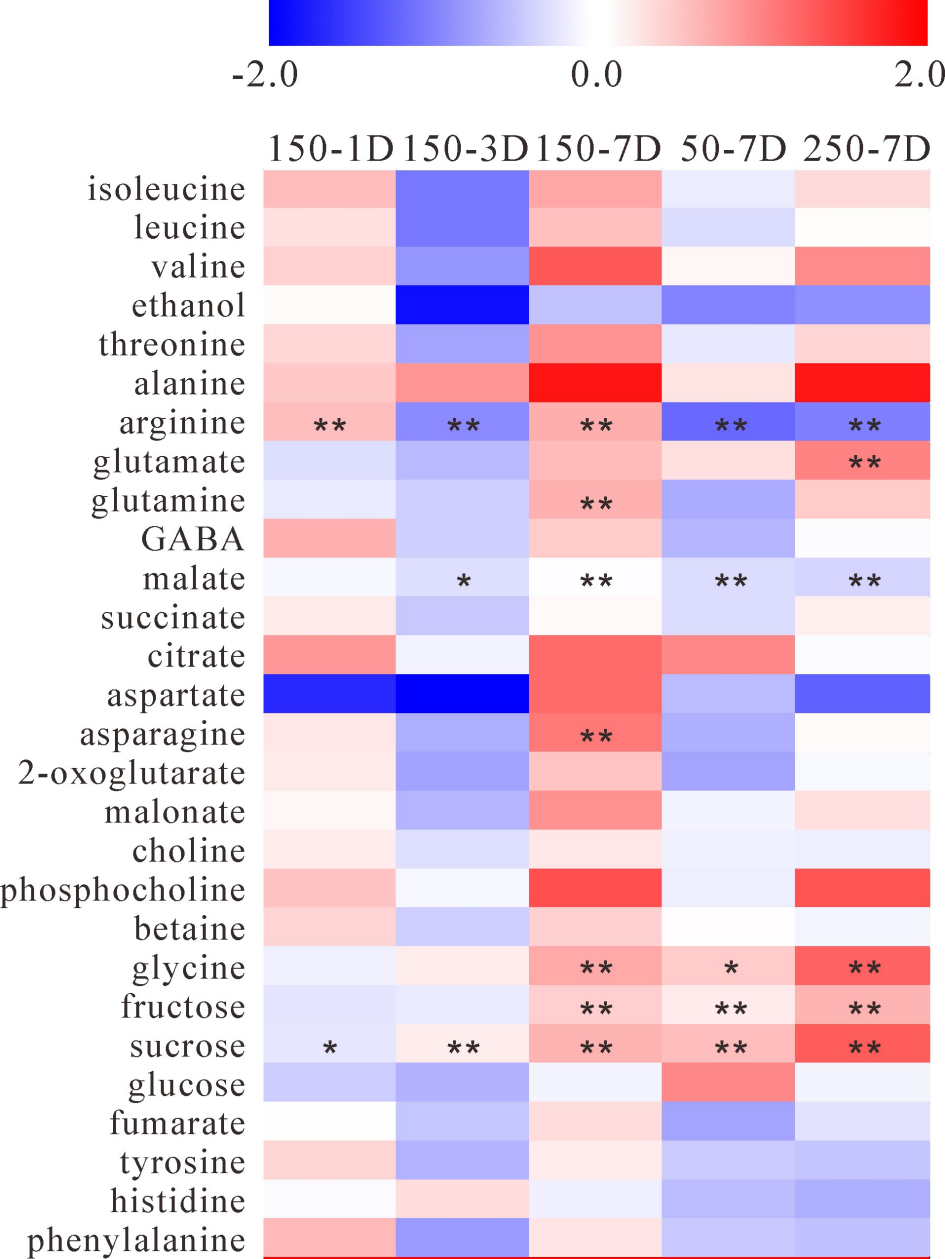
Heat-map of metabolic changes in Jerusalem artichoke tubers under different salt stress. Intensity of colors represents log_2_-transformed ratios of measured means (n = 10) of an analyte’s intensity to its respective mean value in the control conditions. Asterisks mark *t*-test *P*-value, where “**” marks *P* < 0.01 and “*” marks *P* < 0.05.

Jerusalem artichoke tubers under moderate salt stress for 1 day remained unsprouted, resulting in relatively minor effects on the metabolic status (Fig 5), in accordance with the results of PCA, where no significant difference was observed between the untreated and 1-day salt-treated groups (Fig 3). The content of nearly all metabolites significantly increased in 7-day moderate salt-treated Jerusalem artichoke tubers relative to those in the control tubers. However, as shown in Fig 1, sprouting was severely inhibited in Jerusalem artichoke under severe salt stress, indicating its moderate tolerance to salinity, which agrees well with the results of previous studies [4,6]. Furthermore, metabolomics analysis was also performed on Jerusalem artichoke tubers under severe salt stress for 7 days, as well as those under mild salt stress (50 mM NaCl). As a result, the number of metabolites exhibiting a positive fold change in the salt treatment group relative to the control group was almost the same as that of metabolites exhibiting a negative fold change under severe salt stress, while more metabolites, though only moderately, exhibited a negative fold change under mild salt stress, with both salt levels showing quite different response patterns (Fig 5).

#### Carbohydrates

Moderate salt stress facilitated the accumulation of fructose, from a 0.95- to 1.42-fold increase relative to that in the control plants during sprouting (Fig 5 and S1 Table). Additionally, the response pattern of sucrose was also shown to be time-dependent under moderate salt stress, with the level increasing by a 0.96- to 1.62-fold with time, indicating the essential role of sucrose in tuber respiration and sprouting (Fig 5 and S1 Table). These results reflect an increase in the degree of depolymerization of inulin, as sprouting is one factor leading to the remobilization of storage compounds in tuber plants [23], especially when they are subjected to salt stress. Moreover, both mild (50 mM NaCl) and severe salt treatment in the present study induced the accumulation of fructose and sucrose, which both showed NaCl concentration-dependent response patterns to salt stress.

Before Jerusalem artichoke becomes photoautotrophic, shoot and root development relies on nutritional supplies from their mother tubers, including the mobilization of storage reserves to provide energy and carbon. The fold change in glucose content fluctuated during the developmental stage of sprouting in the present study (Fig 5 and S1 Table).

#### Amino acids

Although most detected metabolites in the moderate salt stress group had little response in terms of fold change in the salt-treated plants relative to those in the control on the first day, aspartate showed a dramatic decrease (Fig 5 and S1 Table). Moreover, aspartate surprisingly accumulated with over a 2-fold increase after Jerusalem artichoke became autotrophic, as did asparagine. However, the opposite fold change was observed for Jerusalem artichoke tubers under mild and severe salt stress. Because metabolism was inhibited to a great extent by severe salt stress, the accumulation of aspartate was likely suppressed. Mild salt stress did not enhance the accumulation of most metabolites, including aspartate.

Moderate salt stress suppressed the accumulation of nearly all metabolites at the start of sprouting (3 days) (Fig 5). The only exception was alanine, whose accumulation increased continuously during the period of moderate salt treatment, exhibiting a 1.4- to 3.6-fold increase (Fig 5 and S1 Table). Furthermore, severe but not mild salt stress accelerated the accumulation of alanine to nearly the same extent as moderate salt stress. Salt stress induced significant changes in amino acid metabolism, which resulted in the enhanced accumulation of alanine in Jerusalem artichoke tubers.

In addition, GABA was quickly consumed prior to sprouting, the content of which dramatically increased at the start of sprouting and decreased after the plant became autotrophic, exhibiting fluctuation during the developmental stage of sprouting (S1 Table). However, the GABA content in Jerusalem artichoke tubers under moderate salt stress relative to that in tubers in the control group was found to be almost constant and was maintained at the level in tubers before salt stress (Fig 5). Hence, it seems that GABA was dual-regulated in different developmental stages of sprouting by moderate salt stress. For example, salt stress enhanced GABA accumulation in Jerusalem artichoke tubers either prior to sprouting or after the autotrophic stage.

#### Other metabolites

Six intermediate metabolites involved in the citrate cycle were detected in the present study (Fig 2). Before sprouting, moderate salt stress had little effect on these metabolites except citrate, whose accumulation was significantly enhanced by 1.85-fold (Fig 5). At the start of sprouting, the accumulation of these metabolites was moderately repressed, which was enhanced again after Jerusalem artichoke became autotrophic, especially for citrate and malonate. Moreover, the citrate/malate ratio in tubers under moderate salt stress was 0.10–0.11 during salt treatment, while for control plants, this ratio was 0.05–0.10 (S1 Table), indicating that moderate salt stress likely reduced fluctuations in the citrate/malate ratio. In addition, the citrate/malate ratio decreased dramatically to 0.06 under severe salt stress.

## Discussion

For tuber plants, emerging sprouts have to subsist on carbohydrates and amino acids provided by their mother tubers, and this dependency can continue for some time after emergence, allowing for sustained growth until the plants become autotrophic [24]. Moderate salt stress seems to have a negative effect on the initiation of sprouting of Jerusalem artichoke tubers, as the level of most metabolites decreased relative to that of metabolites in the control plants (Fig 5 and S1 Table), suggesting the downregulation of metabolism in cells at the start of sprouting under salt stress. However, the accumulation of nearly all metabolites accelerated in moderate salt-treated Jerusalem artichoke tubers, when they became autotrophic. Together with the results observed in mild and severe salt-treated Jerusalem artichoke tubers, we assumed that only an appropriate saline environment may trigger metabolism in Jerusalem artichoke tubers as soon as plants become autotrophic.

As mentioned in our previous proteomic analysis, carbohydrate metabolism was activated in Jerusalem artichoke under salt stress, where several enzymes involved in the citrate cycle, glycolysis and pentose phosphate pathways had higher abundances [22]. According to our transcriptome analysis, several genes encoding enzymes in the glucan metabolic pathway were remarkably induced by NaCl treatment, catalyzing the cleavage of individual glucosyl residues from glucans into their monomers [22]. Furthermore, sprouting in Jerusalem artichoke tubers is associated with a dramatic increase in the fructose content [25]. Previous studies have demonstrated that various storage carbohydrates in tubers offer Jerusalem artichoke seedlings the capacity to thrive under environmental stresses, such as drought and salt stress [11,12]. Soluble carbohydrates might play a key role as osmolytes and energy resources in salt defense, which has been demonstrated to be especially important in halophyte species [26]. It could be explained that glucose was mainly consumed for energy and further incorporated into oligosaccharides and polysaccharides in cells, playing an important physiological role at the start of sprouting in Jerusalem artichoke tubers under salt stress, while it increased in abundance as soon as plants became autotrophic (7 days). Similar to fructose, accumulation of the monomer saccharide glucose might have also helped plants adapt to a saline environment. Intriguingly, a significant further accumulation of glucose was only observed in Jerusalem artichoke under mild salt stress, while no differences were observed between the salinity-treated and control groups under both moderate and severe salt stress levels. Therefore, it is speculated that more photosynthesized glucose would be consumed under higher salinity stress, e.g., 150 mM and 250 mM NaCl. In other words, more glucose was required for the accumulation of more metabolites under moderate or severe salt stress levels in the present study.

Both malate and citrate are usually used as mobile energy sources in plants, especially when carbohydrates are depleted [27,28]. The dramatic decrease in the citrate/malate ratio under severe salt stress indicated that respiration and photosynthesis were partly impaired. The decreased fluctuations in the citrate/malate ratio in sprouting Jerusalem artichoke tubers under moderate salt stress could constitute an important element of their adaptive strategy to these conditions.

Aspartate, the initial product of photosynthesis, is used in sugar formation [29] and surprisingly accumulated after Jerusalem artichoke became autotrophic. We therefore assumed that photosynthesis may be enhanced in Jerusalem artichoke tubers under moderate salt stress. Alanine is derived from the glycolysis intermediate pyruvate in a single transamination step. In addition to playing important roles in the control of cytoplasmic pH [30], alanine synthesis saves C3 skeletons, avoiding a shortage in carbon availability [31]. In addition to alanine, GABA can also rapidly re‐enter the citric acid cycle upon reoxygenation, minimizing the net loss of carbon [31]. Clausen *et al*. assumed that GABA may serve as a carbon and nitrogen reserve during sprouting in Jerusalem artichoke tubers [32,33]. All these metabolic responses would help Jerusalem artichoke tubers mitigate the potentially damaging consequences caused by salt stress, resulting in a protective effect.

This study provides a metabolomic investigation of sprouting Jerusalem artichoke tubers under different salt stress levels. Our results suggest that some mechanisms are involved in the acclimation of Jerusalem artichoke tubers to high-salinity environments, including the activation of carbohydrate metabolism and the enhancement of photosynthesis.

## Supporting information

S1 Table The content of the main metabolites in Jerusalem artichoke samples after salt treatment (μmol g^-1^). (DOCX)
