# Supplementary material for: Salt stress induced differential metabolic responses in the sprouting tubers of Jerusalem artichoke (Helianthus tuberosus L.)
Source: PLoS One. 2020 Jun 29;15(6):e0235415. doi: 10.1371/journal.pone.0235415 (PMC7323981; doi:10.1371/journal.pone.0235415)
Supplement: S1 Table — (DOCX) [file pone.0235415.s001.docx]

**Table S1**. The content of the main metabolites in Jerusalem artichoke samples after salt treatment (μmol g^-1^).

|  |  | CK0 D | CK1 D | CK3 D | CK7 D | 150-1 D | 150-3 D | 150-7 D | 50-7 D | 250-7 D |
| --- | --- | --- | --- | --- | --- | --- | --- | --- | --- | --- |
| 1 | isoleucine | 0.3204±  0.0246 | 0.2441±  0.0210 | 0.6667±  0.1048 | 0.2894±  0.0164 | 0.3778±  0.0257 | 0.3316±  0.0191 | 0.5020±  0.0476 | 0.2857±  0.0190 | 0.3851±  0.0422 |
| 2 | leucine | 0.4019±  0.0176 | 0.3657±  0.0269 | 0.8401±  0.1335 | 0.4509±  0.0256 | 0.4744±  0.0441 | 0.4182±  0.0145 | 0.6898±  0.0661 | 0.4063±  0.0304 | 0.5068±  0.0444 |
| 3 | valine | 0.2950±  0.0146 | 0.2153±  0.0132 | 0.5007±  0.0823 | 0.2250±  0.0108 | 0.3001±  0.0199 | 0.2968±  0.0166 | 0.5788±  0.0565 | 0.2599±  0.0105 | 0.4442±  0.0563 |
| 4 | ethanol | 0.1523±  0.0208 | 0.2899±  0.1318 | 0.6131±  0.1329 | 0.2796±  0.1094 | 0.32754±  0.0632 | 0.1619±  0.0440 | 0.2154±  0.0345 | 0.1484±  0.0267 | 0.1591±  0.0239 |
| 5 | threonine | 0.5211±  0.0264 | 0.3278±  0.0218 | 0.6627±  0.1099 | 0.3678±  0.0218 | 0.4446±  0.0279 | 0.4283±  0.02048 | 0.7044±  0.0627 | 0.3557±  0.0187 | 0.5020±  0.0508 |
| 6 | alanine | 0.4377±  0.0249 | 0.4515±  0.0211 | 0.3585±  0.0485 | 0.1690±  0.0121 | 0.6545±  0.0331 | 0.6757±  0.0659 | 0.6133±  0.0707 | 0.2147±  0.0141 | 0.6030±  0.0664 |
| 7 | arginine | 5.1501±  0.4563 | 2.8358±  0.3881 | 4.8371±  1.1266 | 3.1070±  0.6311 | 4.3485±  0.3881 | 2.7024±  0.4799 | 5.1634±  0.6647 | 1.4129±  0.2064 | 1.6122±  0.2801 |
| 8 | glutamate | 2.4817±  0.1756 | 1.7231±  0.2141 | 1.5186±  0.1492 | 1.0118±  0.0908 | 1.5652±  0.3419 | 1.1126±  0.0929 | 1.5741±  0.0876 | 1.3107±  0.0758 | 2.0888±  0.1975 |
| 9 | glutamine | 6.5079±  0.3389 | 1.9774±  0.1442 | 2.3456±  0.2747 | 1.6501±  0.1315 | 1.9396±  0.1121 | 1.9436±  0.2098 | 2.7178±  0.1488 | 1.1133±  0.0828 | 2.3752±  0.1999 |
| 10 | GABA | 1.7521±  0.1339 | 1.0160±  0.0516 | 1.7668±  0.4379 | 1.0932±  0.0775 | 1.6707±  0.1979 | 1.4803±  0.1626 | 1.5761±  0.1974 | 0.7819±  0.0791 | 1.1903±  0.2040 |
| 11 | malate | 8.2059±  0.6482 | 14.7185±  1.0407 | 12.9665±  1.6203 | 10.1973±  0.4897 | 15.5411±  1.2570 | 11.7750±  1.3233 | 11.1404±  1.1668 | 9.1859±  0.5424 | 8.7987±  0.9235 |
| 12 | succinate | 0.2295±  0.0163 | 0.3373±  0.0225 | 0.4163±  0.0643 | 0.3010±  0.0145 | 0.4147±  0.0307 | 0.3331±  0.0252 | 0.3430±  0.0492 | 0.2702±  0.0220 | 0.3611±  0.0346 |
| 13 | citrate | 0.7157±  0.0930 | 0.8971±  0.1072 | 1.3008±  0.2392 | 0.4658±  0.0782 | 1.6581±  0.3159 | 1.3433±  0.1374 | 1.0959±  0.0503 | 0.9390±  0.1334 | 0.5068±  0.0702 |
| 14 | aspartate | 1.2988±  0.1315 | 1.2685±  0.2073 | 1.3222±  0.2256 | 0.4400±  0.0871 | 0.3943±  0.0572 | 0.3227±  0.0874 | 1.0334±  0.3603 | 0.3253±  0.0434 | 0.1910±  0.0411 |
| 15 | asparagine | 3.6057±  0.2757 | 1.6057±  0.1143 | 3.0878±  0.5453 | 1.9722±  0.0762 | 2.0065±  0.1350 | 2.1248±  0.1994 | 4.2934±  0.4201 | 1.3619±  0.0777 | 2.2287±  0.2376 |
| 16 | 2-oxoglutarate | 0.9153±  0.0596 | 0.5147±  0.0514 | 0.8220±  0.1535 | 0.3734±  0.0575 | 0.6331±  0.0683 | 0.5260±  0.0539 | 0.5533±  0.0740 | 0.2418±  0.0256 | 0.3975±  0.0910 |
| 17 | malonate | 0.1318±  0.0082 | 0.1054±  0.0113 | 0.1286±  0.0218 | 0.0651±  0.0067 | 0.1211±  0.0102 | 0.0917±  0.0073 | 0.1254±  0.0136 | 0.0677±  0.0043 | 0.0847±  0.0133 |
| 18 | choline | 0.6189±  0.0337 | 0.5607±  0.0308 | 0.5133±  0.0926 | 0.3731±  0.0178 | 0.6806±  0.0385 | 0.4704±  0.0477 | 0.4653±  0.0660 | 0.3778±  0.0213 | 0.3729±  0.0766 |
| 19 | phosphocholine | 0.1343±  0.0090 | 0.1187±  0.0046 | 0.1314±  0.0120 | 0.0687±  0.0100 | 0.1777±  0.0111 | 0.1384±  0.0079 | 0.1846±  0.0167 | 0.0689±  0.0040 | 0.180±  0.0185 |
| 20 | betaine | 2.2927±  0.1099 | 2.7751±  0.1512 | 3.4066±  0.4095 | 1.9953±  0.1282 | 3.8115±  0.1904 | 2.8295±  0.2023 | 2.8006±  0.2547 | 2.2267±  0.1094 | 2.0840±  0.2222 |
| 21 | glycine | 3.8747±  0.2122 | 4.1585±  0.2313 | 2.8918±  0.3555 | 1.8938±  0.1374 | 4.2038±  0.2592 | 3.5026±  0.2233 | 3.2269±  0.3525 | 2.7241±  0.1475 | 4.6316±  0.5407 |
| 22 | fructose | 7.5930±  0.3142 | 8.2800±  1.1977 | 7.7638±  1.2445 | 5.4393±  0.6496 | 7.8561±  0.8196 | 7.6515±  0.6804 | 7.6991±  0.7899 | 6.6717±  0.7553 | 8.7908±  0.9838 |
| 23 | sucrose | 24.2573±  1.2653 | 28.4517±  2.9442 | 17.4312±  3.2379 | 11.7689±  0.6054 | 27.1854±  2.9155 | 21.1344±  1.6362 | 19.0117±  1.8747 | 18.1257±  0.8587 | 29.7219±  3.3001 |
| 24 | glucose | 0.7437±  0.1504 | 1.1619±  0.1050 | 2.0932±  0.4514 | 0.7655±  0.1265 | 0.9560±  0.0590 | 1.4711±  0.2414 | 0.7949±  0.1304 | 1.5370±  0.1023 | 0.7943±  0.1008 |
| 25 | fumarate | 0.2926±  0.0265 | 0.2397±  0.0284 | 0.2748±  0.0406 | 0.2064±  0.0212 | 0.2671±  0.0261 | 0.2157±  0.0221 | 0.2713±  0.0454 | 0.1336±  0.0084 | 0.1926±  0.0450 |
| 26 | tyrosine | 0.1004±  0.0146 | 0.0876±  0.0086 | 0.1517±  0.0206 | 0.1142±  0.0102 | 0.1194±  0.0075 | 0.1065±  0.0105 | 0.1384±  0.0180 | 0.0929±  0.0082 | 0.0891±  0.0135 |
| 27 | histidine | 0.1253±  0.0719 | 0.0940±  0.0092 | 0.0882±  0.0170 | 0.0940±  0.0326 | 0.1017±  0.008 | 0.1156±  0.0539 | 0.0947±  0.0083 | 0.0699±  0.0035 | 0.0647±  0.0089 |
| 28 | phenylalanine | 0.32108±  0.0121 | 0.3622±  0.0156 | 0.7345±  0.1102 | 0.3787±  0.0159 | 0.5692±  0.0324 | 0.4480±  0.0322 | 0.4779±  0.0370 | 0.3042±  0.0169 | 0.2911±  0.0300 |
